# 355. Stratification of Patient-Specific Risk Factors for Multidrug-Resistant Gram-Negative Respiratory Pathogens in Intensive Care Units (ICU)

**DOI:** 10.1093/ofid/ofad500.426

**Published:** 2023-11-27

**Authors:** Walaiporn Wangchinda, Samuel L Aitken, Paul Lephart, jason M Pogue

**Affiliations:** University of Michigan College of Pharmacy, Ann Arbor, MI; Michigan Medicine, Ann Arbor, Michigan; University of Michigan, Ann Arbor, Michigan; University of Michigan, College of Pharmacy, Ann Arbor, Michigan

## Abstract

**Background:**

Guidelines for nosocomial pneumonia recommend empiric combination therapy for patients with a risk of infection due to multidrug-resistant Gram-negative pathogens in ICUs. Common risk factors cited include ≥ 5-day duration in the ICU, transfer from other healthcare facilities, recent antibiotic use, and history of a resistant Gram-negative pathogen. However, the interrelation of these factors and which are key drivers remain ill-defined. This study is designed to stratify risk factors to further optimize empiric regimens.

**Methods:**

A total of 190 patients who had a positive Gram-negative respiratory culture obtained from the MICU/SICU of Michigan Medicine in 2021 were included. Patients were classified into subgroups based on the presence/absence of each aforementioned risk factor. Cumulative susceptibility percentages of isolates in each risk group were examined and compared.

**Results:**

Cumulative susceptibility percentages of Gram-negative respiratory isolates from patients with/without each risk factor are shown in Figure 1. Patients with recent antibiotic use or history of a resistant Gram-negative pathogen had noticeably lower susceptibility rates than those without those risk factors, whereas the presence/absence of prolonged ICU stay and/or admission from another facility had minimal impact on susceptibility rates. In patients without recent antibiotic exposure or history of a resistant pathogen, 90% had pathogens that demonstrated *in vitro* susceptibility to cefepime monotherapy. (Figure 2) In contrast, the susceptibility percentages of any β-lactam monotherapy were < 75% in patients with one or more of these factors and remained < 85% when adding an aminoglycoside or fluoroquinolone. More stratified analyses demonstrated that patients with previous positive carbapenem-resistant *P. aeruginosa* or Enterobacterales should receive a novel agent. Proposed stratified empiric regimens are summarized in Figure 3.

**Figure:**
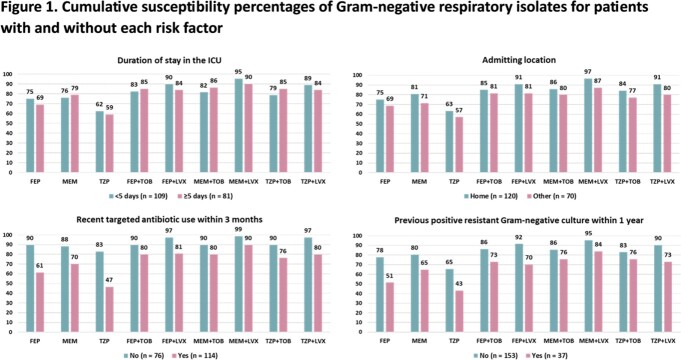

**Figure:**
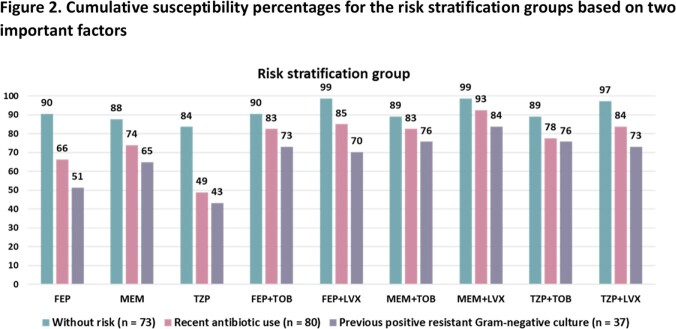

**Figure:**
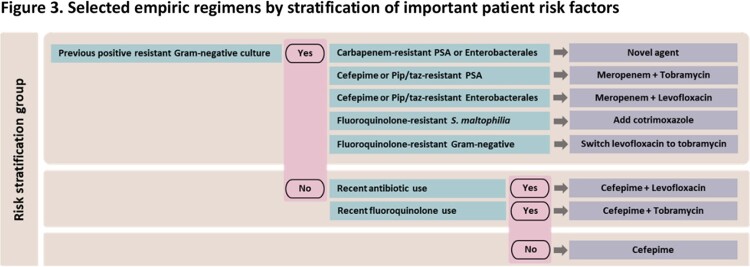

**Conclusion:**

Recent antibiotic use and previous positive resistant Gram-negative cultures were significant factors for determining antibiotic resistance in Gram-negative respiratory pathogens. Risk stratification based on these two factors can help further optimize empiric antibiotic therapy.

**Disclosures:**

**jason M. Pogue, PharmD**, AbbVie: Advisor/Consultant|Entasis: Advisor/Consultant|Ferring: Advisor/Consultant|GSK: Advisor/Consultant|Merck: Advisor/Consultant|Merck: Grant/Research Support|Qpex: Advisor/Consultant|Shionogi: Advisor/Consultant

